# Impacts of Five Different Drying Methods on Volatile Organic Compounds in Mulberry Fruits

**DOI:** 10.3390/foods13213514

**Published:** 2024-11-02

**Authors:** Xinyi Yin, Wenxi Xiao, Shijia Zhang, Ziran Yu, Wen Ai, Shasha Fu, Jianjun Liu, Dan Huang

**Affiliations:** State Key Laboratory of Chinese Medicine Powder and Medicine Innovation in Hunan (Incubation), Science and Technology Innovation Center, Hunan University of Chinese Medicine, Changsha 410208, China; yxy@stu.hnucm.edu.cn (X.Y.); xiaowenxi@stu.hnucm.edu.cn (W.X.); zhangshijia@stu.hnucm.edu.cn (S.Z.); yuziran@stu.hnucm.edu.cn (Z.Y.); aiwen@stu.hnucm.edu.cn (W.A.); fushasha@stu.hnucm.edu.cn (S.F.); liujianjun2018@hnucm.edu.cn (J.L.)

**Keywords:** mulberry fruit, GC-IMS, volatile organic compounds, drying methods, PCA, CA, PLS-DA

## Abstract

The mulberry fruit is edible and medicinal, and it is commonly referred to as the “best health product of the 21st century”. The purpose of this study was to find out whether different drying methods affect the quality of mulberry fruits and the main nature of the volatile organic compounds (VOCs) they contain. This study used vacuum freeze-drying (VFD), vacuum drying (VD), sun drying (SD), hot-air drying (HAD), and microwave drying (MD) to treat fresh mulberry fruits. Gas-phase ion mobility spectrometry (GC-IMS) was used to detect and analyze the VOCs in mulberry fruit samples treated with the different drying methods. There were 47 VOCs detected, with aldehydes and alcohols dominating. The obtained data were subjected to principal component analysis (PCA), cluster analysis (CA), nearest neighbor fingerprint analysis, and partial least-squares regression analysis (PLS-DA). The conclusion was drawn that fresh mulberry fruits contain abundant VOCs, and mulberry fruits after VD contain many aldehydes; thus, VD promoted the synthesis of phellandrene and other compounds widely used in the preparation of cosmetics such as perfume and soap. HAD promoted the synthesis of esters commonly used in the preparation of fruit flavor and wine essence. The higher (*E*)-2-heptenal content with SD was conducive to the Maillard reaction. MD promoted the synthesis of heptanal and valeraldehyde with aroma characteristics such as fatty, green, fruity, grassy, and floral. According to the VIP results, VOCs (*E*)-2-heptenal, pentanal D, cyclohexanone, and 2-hexanone D influenced the VOCs in most of the mulberry fruit samples. The findings of this study provide an important reference for drying mulberry fruits, which, in turn, will help to ensure the safety and effectiveness of processed mulberry fruit products.

## 1. Introduction

Mulberry fruit is the fleshy fruit-spike of *Morus alba* L., harvested from April to June when the fruit turns red or black [1]. In addition to being rich in vitamins and other nutrients that maintain human metabolism, mulberry fruit contains various functional components, such as polysaccharides, polyphenols, alkaloids, and volatile organic compounds (VOCs). It has high nutritional and health value and is known as the “best health product of the 21st century” [2,3]. Researchers have confirmed that mulberry fruit improves body immunity, prevents cancer and mutations, protects the liver and kidney, resists oxidation, delays cell aging, promotes the growth of hematopoietic cells, reduces blood glucose and lipids, and alleviates diabetes symptoms [4,5,6,7]. The polysaccharides and VOCs in mulberry fruit possess strong antioxidant properties in vitro and in vivo [8,9], and significantly influence its taste.

Fresh mulberry fruit is susceptible to spoilage due to its high water and sugar contents and short season. To address these issues, researchers have focused on developing products using mulberry fruit as the raw material, including mulberry fruit pulp, mulberry fruit wine, mulberry fruit jam, and mulberry fruit candy, or storing them after drying.

Food drying is a widely used method of preserving and processing food. Drying fresh fruits maintains their quality and nutrient content and extends their shelf life [10,11]. The traditional mulberry fruit processing method is to directly sun dry or steam the harvested fresh fruits before sun drying. Studies have shown that drying methods affect different nutrients in the same food to varying degrees [12,13]. Odor is an important evaluation indicator in the traditional quality evaluation system for food, and VOCs are an important basis for distinguishing food quality [14,15]. It is currently unknown how different drying methods and temperatures affect the chemical composition and pharmacological properties of mulberry fruits.

Gas chromatography–ion mobility spectrometry (GC-IMS), which combines gas chromatography with ion mobility spectrometry, is a newly emerging technology that enables fast odor analysis through the use of gas chromatography and ion mobility spectrometry. It is possible to detect and analyze VOCs under normal pressure conditions, and the technology is portable, easy to use, and fast in detecting VOCs. It is an ideal tool for analyzing spices and flavors in food [16,17,18,19].

This study was conducted to discover and analyze VOCs in five different drying methods (vacuum freeze-drying (VFD), vacuum drying (VD), microwave drying (MD), sun drying (SD), and hot-air drying (HAD)) and fresh mulberry fruits in order to provide quality reference data for the evaluation and processing of mulberry fruit products.

## 2. Materials and Methods

### 2.1. Materials

The mulberry fruits were collected from Sichuan, China.

### 2.2. Drying Procedures

The method of Peng et al. was followed, with some minor amendments [19]. Fresh mulberry fruits were dried to a constant weight. VFD was performed on 500 g of fresh mulberry fruits for 10 h using a freeze-dryer. VD was performed on 500 g of fresh mulberry fruit for 10 h at 60 °C. HAD was performed on 500 g of fresh mulberry fruits at 60 °C with an air velocity of 0.4 m/s for 10 h. MD was performed on 500 g of fresh mulberry fruit in a microwave oven and dried 3 times for 1.5 h each time. SD was performed on 500 g of fresh mulberry fruits by exposing them to natural sunlight for 3 days, 10 h a day, on stainless-steel trays. The dried mulberry fruits were then ground into powder for analysis.

### 2.3. GC–IMS Analysis

A FlavorSpec^®^ gas-phase ion mobility spectrometer from G.A.S. (Dortmund, Germany) was used to directly analyze the VOCs in the dried powders. The GC-IMS was analyzed by the method of Peng et al. [19].

The two-dimensional top view and different images of the gas-phase ion migration spectra were combined in this study in order to analyze the spectra in detail.

### 2.4. Statistical Analysis

Plugins such as Reporter, Gallery Plot, and Dynamic PCA in VOCal data processing software (from G.A.S., Dortmund, Germany, version 2.0.0) were used to analyze the three-dimensional spectra, two-dimensional spectra, fingerprints, “nearest neighbor” fingerprints, and PCA of VOCs. “Nearest neighbor” fingerprint analysis, based on fingerprint similarity map analysis, is a distance-based clustering analysis method used to evaluate differences between samples [20]. PCA is widely used to reduce data dimensionality by linearly transforming multiple variables (features) into a few principal components. These components retain most of the information (variance) in the original data while minimizing correlation (orthogonality). PCA enables the visualization of the model, facilitating easy comprehension and minimizing subjective judgments [21,22,23]. PCA is employed in various fields, including statistical analysis, signal and image processing, and pattern recognition. Cluster analysis (CA) and partial least-squares regression analysis (PLS-DA) were conducted using TBtools and SIMCA (Version 14.1, Umetrics, Sweden), respectively.

## 3. Results

### 3.1. Influences of Different Drying Methods on the Appearance of Mulberry Fruits

**Figure 1:**
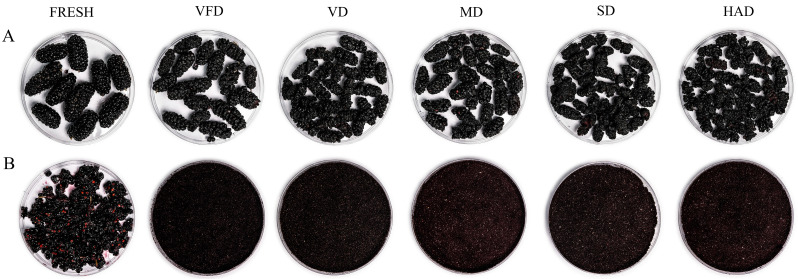
Photos of mulberry fruits (**A**) and powders (**B**) exposed to different drying methods. VFD: vacuum freeze-drying; VD: vacuum drying; MD: microwave drying; SD: sun drying; HAD: hot-air drying.

Figure 1 shows the appearance of the mulberry fruits before and after drying using different methods. The VFD sample was nearly identical to the fresh mulberry fruits in terms of shape, while all other samples had shrunk. Due to the different drying conditions, the mulberry fruits also changed significantly in color. The color of the VFD sample was purple and closest in color and appearance to the fresh mulberry fruits. The MD sample was slightly lighter in color and appearance than the VFD sample, showing a small amount of change. In contrast, the other dried mulberry fruits had significantly changed appearances; MD and SD samples showed the greatest changes in appearance and had the darkest color.

### 3.2. Analysis of GC-IMS Test Results

#### 3.2.1. Comparison of Differences in VOCs in Mulberry Fruits Processed Using Different Drying Methods

**Figure 2:**
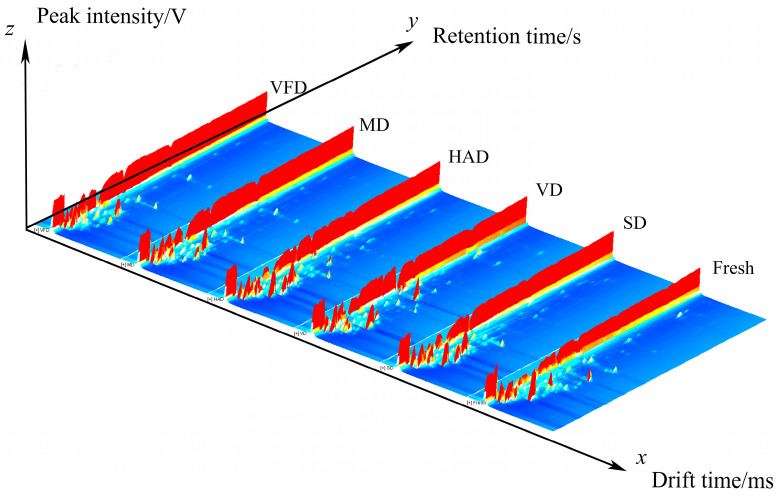
Three-dimensional spectra of VOCs in six groups of mulberry fruit samples (The red protrusion represents the signal of VOCs).

The reporter plugin of the GC-IMS instrument analysis software was used to generate three-dimensional spectra of VOCs in mulberry fruits (Figure 2). Drift time, GC retention time, and peak intensity are represented by the x-, y-, and z-axes, respectively. In the *z*-axis, a peak indicates a volatile component, and its height indicates the amount of the component. Figure 2 shows that the different drying methods resulted in different VOCs in the mulberry fruits.

**Figure 3:**
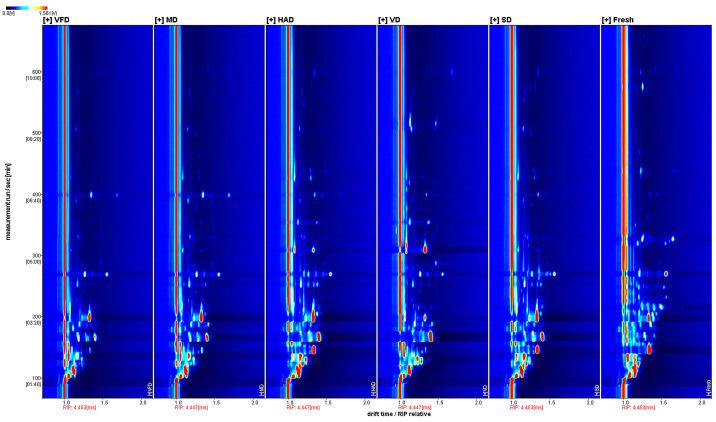
Two-dimensional spectra of VOCs in six groups of mulberry fruit samples.

The gas-phase ion migration spectra of the VOCs in the fresh mulberry fruits and mulberry fruits treated with different drying methods are shown in Figure 3, with the horizontal and vertical axes representing the ion migration and retention times, respectively. Bright spots represent volatile components and red vertical lines indicate reactive ions. A white component represents a lower content, while a red component represents a higher content. Figure 3 presents a visual comparison between the VOCs present in the dried mulberry fruit samples treated with the different drying methods.

**Figure 4:**
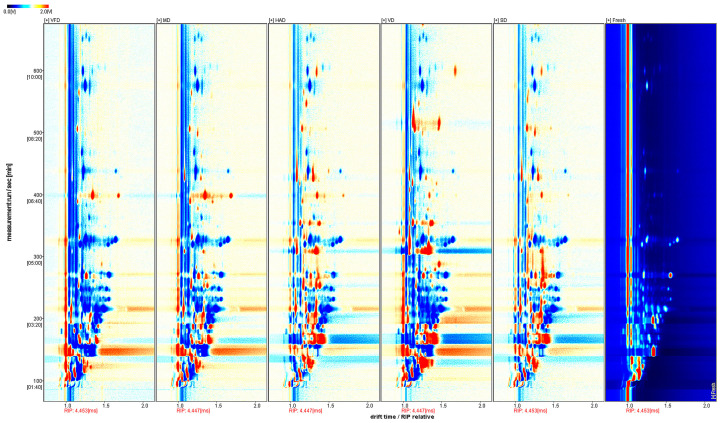
Spectral comparison of FRESH fruit samples with the other five groups.

Figure 4 shows a comparison chart using the fast response and enhanced sensitivity for the heterogeneous materials (FRESH) spectrum as a reference. A white background indicates that the target and reference samples have the same VOC content; in red, the concentration in the target sample is greater than in the reference; in blue, the concentration is lower.

#### 3.2.2. GC-IMS Analysis of VOCs in Mulberry Fruits Treated with Different Drying Methods

GC retention index (NIST 2020) and IMS migration time databases were retrieved and compared in this study. In the six mulberry fruit samples, 47 VOCs were detected, including 18 aldehydes, accounting for approximately 38.3%; seven alcohols, accounting for approximately 14.9%; six ketones, accounting for approximately 12.8%; and five terpenes, including furan, ester compounds, etc. The results are shown in Table 1 (the substance suffixes M, D, and P represent monomers, dimers, and polymers of the same substance, respectively).

#### 3.2.3. GC-IMS Fingerprint Analysis of VOCs in Mulberry Fruits Treated with Different Drying Methods

**Figure 5:**
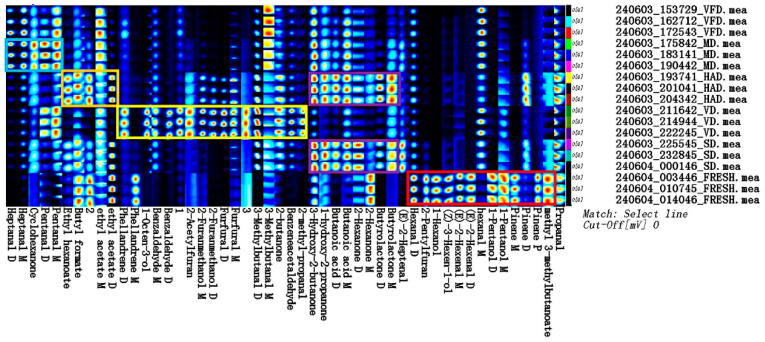
Fingerprint analysis of VOCs in mulberry fruit samples.

Fingerprint analysis was used to compare the effects of different drying methods on the VOCs released from mulberry fruits (Figure 5). The spot intensities of the different VOCs in the samples can be determined in the fingerprint spectra, thereby revealing the differences in the concentrations of the different substances [24,25]. The results of the comparative analysis of the VOCs in the FRESH, SD, VFD, MD, HAD, and VD samples show that the MD sample contained higher contents of heptanal D, heptanal M, cyclohexanone, pentanal D, and pentanal M, as shown in the blue box. As shown in the orange box, the HAD sample contained higher contents of butyl formate, ethyl hexanoate, and ethyl acetate (ethyl acetate D and ethyl acetate M). As shown in the yellow box, the VD sample contained relatively high contents of phellandrene D, 1-octen-3-ol, benzaldehyde D, benzaldehyde M, 2-acetylfuran, 2-furanmethanol D, 2-furanmethanol M, furfural D, furfural M, 3-methylbutanal D, 3-methylbutanal M, 2-butanone, benzaldehyde, and 2-methylpropanal. As shown in the purple box, HAD and SD samples contained higher contents of 3-hydroxy-2-butanone, 1-hydroxy-2-propanone, butanoic acid M, butanoic acid D, 2-hexanone D, and butyrolactone D. As shown in the red box, the FRESH sample contained higher contents of hexanal D, 1-hexanol, 2-pentylfuran, 3-hexen-1-ol, 2-hexenal M, 2-hexenal D, 1-pentanol M, pinene M, and methyl 3-methylbutanoate.

### 3.3. Chemometric Analysis

Chemometrics applies mathematics, statistics, and other methods (including computers) to select the optimal experimental design and measurement methods and obtains qualitative, quantitative, morphological, structural, and other information about substances to the maximum extent possible by processing and analyzing measurement data. The three most commonly used chemometric methods in GC-IMS data analysis are PCA, CA, and PLS-DA. PCA is commonly used for model recognition to observe sample similarity and is combined with CA and PLS-DA to classify samples based on their features. In traditional Chinese medicine, agricultural products, food classification, and other fields, these methods are widely used.

#### 3.3.1. Principal Component Analysis (PCA)

**Figure 6:**
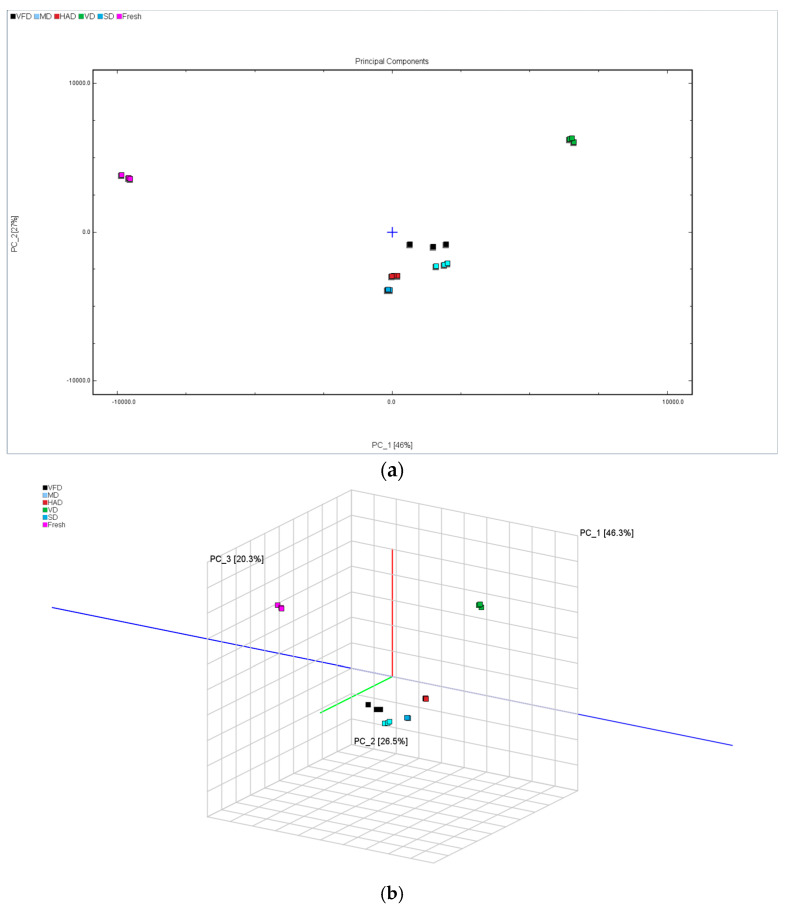
Plot of PCA scores of VOCs in six groups of mulberry fruit samples. (**a**) PCA score plot; (**b**) three-dimensional scatter plot.

The principal components of the VOCs in the FRESH, SD, VFD, MD, HAD, and VD samples were analyzed using the Dynamic PCA plugin in the VOCal data processing software. Figure 6 shows the results. This study found a cumulative contribution rate of 72.8% for the principal components, with PC1 and PC2 contributing 46.3% and 26.5%, respectively. The graph shows that the distance between SD, HAD, and FRESH was the smallest, indicating that the VOC contents in sun-dried and hot-air-dried mulberry fruits were closest to that in fresh mulberry fruits. Conversely, dried fruits processed using the VFD and MD methods exhibited high similarity in their volatile organic compound content dimension, suggesting that these two methods have similar effects on the VOCs in mulberry fruits. The distance between VD and FRESH was the greatest, indicating that their volatile component contents significantly differed from that of fresh mulberry fruits and suggesting differences in VOC contents between mulberry fruits treated with the different drying methods. Perhaps temperature, vacuum degree, and microwave used during drying all have an impact on volatile components.

#### 3.3.2. “Nearest Neighbor” Fingerprint Analysis

**Figure 7:**
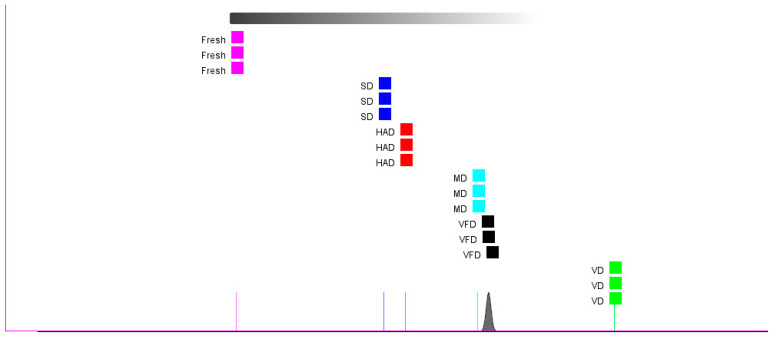
“Nearest neighbor” fingerprint analysis of mulberry fruit samples.

The results of the “nearest neighbor” fingerprint analysis conducted on dried mulberry fruit samples treated with the different drying methods are shown in Figure 7. The distances between SD, HAD, and FRESH are relatively close, while the distance between VD and FRESH is the farthest.

This indicates that the VOCs in dried mulberry fruits treated via SD and HAD were closest to those in fresh mulberry fruits. The VOCs in dried mulberry fruits after VD significantly differed from those in fresh mulberry fruits.

#### 3.3.3. Cluster Analysis (CA)

**Figure 8:**
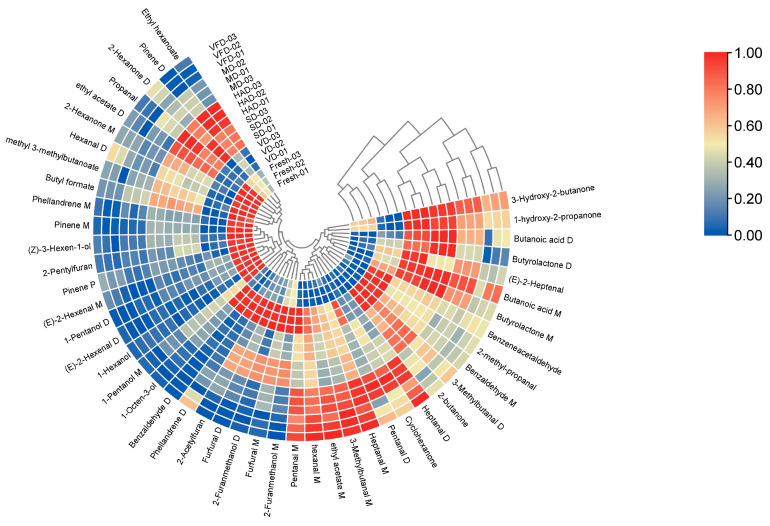
Cluster heatmap of VOCs in six groups of mulberry fruit samples.

Cluster analysis (CA) is widely used to group samples based on their characteristics. It is a non-parametric data interpretation method that is easy to use and can visualize complex data [26,27]. Based on changes in color intensity, the heatmap shows the differences between different groups. The results are shown in Figure 8. SD, HAD, and FRESH samples had relatively similar volatile organic compound contents compared with the other three groups; the SD sample contained higher (*E*)-2-heptenal and 2-hexanone D contents than the other groups; the HAD sample contained higher contents of ethyl hexanoate, propanal, butyrolactone D, and ethyl hexanoate; and SD and HAD samples contained higher and similar contents of 3-hydroxy-2-butanone, pinene D, 1-Hydroxy-2-propanone, butanoic acid M, and butyrolactone M. Simultaneously, VFD and MD samples exhibited significant differences in their volatile component contents compared with FRESH samples. However, the clustering between the two was more obvious, with VD and FRESH samples having the largest difference in volatile component contents.

#### 3.3.4. Partial Least-Squares Discriminant Analysis (PLS-DA)

**Figure 9:**
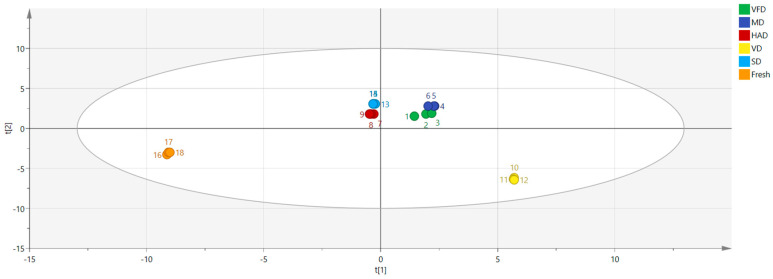
PLS-DA results of VOCs in 6 groups of mulberry fruit samples.

PLS-DA is a statistical method with supervised discriminative patterns, belonging to the model methods, which can effectively explain observed values and suggest corresponding variable predictions [26,27]. The model’s reliability and predictive ability are evaluated according to R^2^ and Q^2^, with R^2^ and Q^2^ values greater than 0.5 indicating an acceptable fit of the model. The closer these values are to one, the stronger the predictive ability. Six sets of sample data, i.e., FRESH, VD, SD, VFD, MD, and HAD, were imported using the SIMCA software. As shown in Figure 9, R^2^X = 0.992, R^2^Y = 0.991, and Q^2^ = 0.971. The distance between HAD and FRESH samples was the smallest, while the other four groups significantly differed from FRESH, consistent with the PCA graph conclusion.

**Figure 10:**
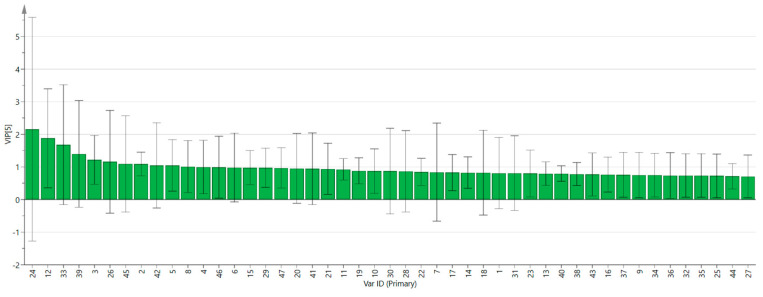
VIP values of the characteristic variables.

**Figure 11:**
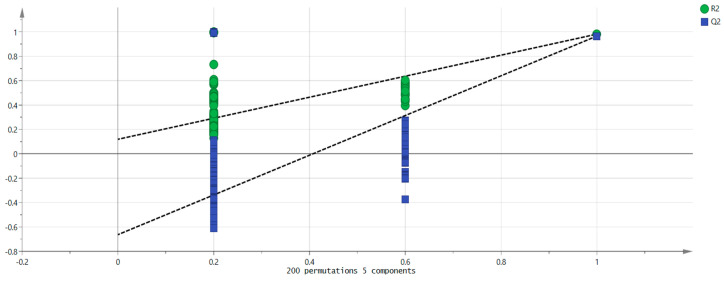
Permutation test results of VOCs in 6 groups of mulberry fruit samples.

In addition, to further measure the contribution of each variable, the PLS-DA model was used to predict the projected importance (VIP) of each volatile component variable. A VIP greater than one indicates that the variable has a high contribution to the overall discriminant model [28,29]. As shown in Figure 10, (*E*)-2-heptenal, pentanal D, cyclohexanone, 2-hexanone D, heptanal D, pinene D, butyrolactone D, heptanal M, ethyl hexanoate, 2-furanmethanol M, and hexanal M were the main indicators of differences. Different types of mulberry fruit samples were distinguished using these compounds as markers. Additionally, 200 cross-validations were conducted simultaneously to examine the R^2^ and Q^2^ values to determine whether the model was overfitting. The graph shows the line with a large slope, indicating that the PLS-DA model was not overfitting (R^2^ = 0.119 and Q^2^ = −0.663, as shown in Figure 11).

## 4. Discussion

In this study, an objective analysis of the odor of mulberry samples was conducted using GC-IMS technology combined with chemical stoichiometry, and the effects of the different drying methods on the VOCs in mulberry fruits were compared. In the mulberry fruit samples, 47 VOCs were identified using this technology. Aldehydes, alcohols, and ketones were the main components of VOCs in mulberry fruits. Using GC-IMS technology to obtain three-dimensional spectra, two-dimensional spectra, and color difference spectra revealed differences in the VOCs between mulberry fruits treated with different drying methods. According to the volatile component fingerprint spectrum results, the FRESH sample contained higher contents of hexanal D, 1-hexynol, 2-pentylfuran, (*Z*)-3-hexen-1-ol, (*E*)-2-hexenal M, etc.; the MD sample contained higher contents of heptanal D, heptanal M, cyclohexanone, pentanal D, and pentanal M; the HAD sample contained higher contents of butyl formate, ethyl hexanoate, ethyl acetate D, and ethyl acetate M; HAD and SD samples contained higher contents of 3-hydroxy-2-butanone, 1-hydroxy-2-proline, butanoic acid M, butanoic acid D, 2-hexanone D, butyrolactone D, and butyrolactone M; and the VD sample contained higher contents of 1-octen-3-ol, benzaldehyde D, and benzaldehyde M.

Based on the research results, it can be concluded that fresh mulberry fruits are rich in VOCs, while VD mulberry fruits have a relatively high content of VOCs. Most aldehydes are present in high concentrations in VD mulberry fruits. The VOCs in dried mulberry fruits are mainly esters, which have a fruity smell and are often used to make fruit and wine essences. SD promotes the synthesis of (*E*)-2-heptanal, which is beneficial for the Maillard reaction [30]. Simultaneously, there are many VOCs with similar contents in both SD and HAD samples, such as 3-hydroxy-2-butanone, which has strong cream, fat, and white peel aromas. It can be directly added to food to add aroma and flavor. MD mulberry fruits contain relatively high levels of two aldehydes: heptanals M and D and 1-pentanol M. These are commonly used fragrances with characteristics such as fatty, light green, fruity and grassy, and floral aromas.

As a result of the PCA, it is confirmed that there were relatively few differences between SD, HAD, and FRESH samples, while VD and FRESH samples were far apart, indicating significant differences. According to the results of the nearest neighbor Euclidean distance graph, the differences between SD, HAD, and FRESH samples were relatively small, while VD and FRESH samples were far apart, indicating that the VOCs in the samples after sun drying and hot-air drying were relatively close to those in fresh mulberry fruits. Meanwhile, the results of the clustering heatmaps and PLS-DA are consistent with the PCA and nearest neighbor fingerprint spectra, further verifying the previous conclusions. According to the VIP results, VOCs such as (*E*)-2-heptenal, pentanal D, cyclohexanone, and 2-hexanone D have the greatest impact on the flavor of mulberry fruits.

## 5. Conclusions

In this study, the VOCs in mulberry fruits treated with five different drying methods were analyzed and compared using GC-IMS combined with chemometric techniques. A total of 47 VOCs were identified, including 18 aldehydes, accounting for approximately 38.3%; seven alcohols, accounting for approximately 14.9%; six ketones, accounting for approximately 12.8%; and five terpenes, accounting for approximately 10.6%. Therefore, aldehydes and alcohols are the most abundant VOCs in mulberry fruits. A fingerprint map was established using the feature components fitted with the Gallery plot plugin software, and it was found that the ester content was higher in the HAD sample, the aldehyde content was higher in the VD and MD samples, and the alcohol content was higher in the FRESH sample. The mulberry fruit samples treated with the different drying methods contained the same VOCs, but their contents varied. Moreover, changing the drying method also affected the VOC contents in the mulberry fruits. The data were visualized by conducting data analyses, such as principal component analysis (PCA), nearest neighbor fingerprint spectra, cluster analysis (CA), and partial least-squares discriminant analysis (PLS-DA), confirming the differences in the VOCs in the mulberry fruits treated with the different drying methods. Research has shown that sun drying and other forms of drying are still feasible drying methods for the industrial production of mulberry fruits.

This study compared the VOCs in mulberry fruit samples treated with different drying methods using GC-IMS combined with chemical stoichiometry. This fast, highly sensitive, and high-resolution technology can quickly and efficiently detect relatively low concentrations of VOCs. There is great potential for its use in the analysis of fragrances, the identification of authenticity, and the control of food quality.

## Figures and Tables

**Table 1:** The components of VOCs in mulberry fruits.

NO	Compound	CAS	Molecular Formula	MW	RI	Rt/s	Dt/ms
1	Phenylacetaldehyde	C122781	C_8_H_8_O	120.2	1053.7	679.007	1.2595
2	Heptanal M	C111717	C_7_H_14_O	114.2	901.2	398.818	1.3518
3	Heptanal D	C111717	C_7_H_14_O	114.2	899.6	396.483	1.6891
4	2-Furanmethanol D	C98000	C_5_H_6_O_2_	98.1	869.5	354.442	1.3830
5	2-Furanmethanol M	C98000	C_5_H_6_O_2_	98.1	871.0	356.388	1.1259
6	Furfural M	C98011	C_5_H_4_O_2_	96.1	837.8	315.126	1.0863
7	Furfural D	C98011	C_5_H_4_O_2_	96.1	834.4	311.233	1.3310
8	Hexanal M	C66251	C_6_H_12_O	100.2	798.4	272.307	1.2695
9	1-Pentanol D	C71410	C_5_H_12_O	88.1	740.1	216.642	1.5017
10	1-Pentanol M	C71410	C_5_H_12_O	88.1	741.5	217.809	1.2518
11	3-Hydroxy-2-butanone	C513860	C_4_H_8_O_2_	88.1	724.4	203.406	1.3330
12	Pentanal D	C110623	C_5_H_10_O	86.1	705.0	188.225	1.4215
13	Pentanal M	C110623	C_5_H_10_O	86.1	704.5	187.836	1.2008
14	3-Methylbutanal D	C590863	C_5_H_10_O	86.1	674.6	168.372	1.4070
15	3-Methylbutanal M	C590863	C_5_H_10_O	86.1	667.4	164.480	1.1873
16	Ethyl acetate D	C141786	C_4_H_8_O_2_	88.1	628.8	145.016	1.3403
17	Ethyl acetate M	C141786	C_4_H_8_O_2_	88.1	628.0	144.627	1.0977
18	2-Methyl-propanal	C78842	C_4_H_8_O	72.1	591.3	128.278	1.2789
19	Butanoic acid M	C107926	C_4_H_8_O_2_	88.1	779.5	253.622	1.1654
20	Butanoic acid D	C107926	C_4_H_8_O_2_	88.1	778.0	252.065	1.3861
21	1-Hydroxy-2-propanone	C116096	C_3_H_6_O_2_	74.1	720.0	199.903	1.2331
22	2-Butanone	C78933	C_4_H_8_O	72.1	598.6	131.392	1.2321
23	Hexanal D	C66251	C_6_H_12_O	100.2	797.8	271.757	1.5586
24	(*E*)-2-Heptenal	C18829555	C_7_H_12_O	112.2	960.4	499.417	1.2548
25	Pinene M	C80568	C_10_H_16_	136.2	926.9	439.735	1.2246
26	Pinene D	C80568	C_10_H_16_	136.2	926.5	439.041	1.2905
27	Pinene P	C80568	C_10_H_16_	136.2	925.6	437.653	1.6595
28	Benzaldehyde D	C100527	C_7_H_6_O	106.1	968.6	515.238	1.4658
29	Benzaldehyde M	C100527	C_7_H_6_O	106.1	964.1	506.489	1.1562
30	1-Octen-3-ol	C3391864	C_8_H_16_O	128.2	969.2	516.421	1.1309
31	Phellandrene D	C99832	C_10_H_16_	136.2	1011.8	599.437	1.6775
32	Phellandrene M	C99832	C_10_H_16_	136.2	1011.1	598.313	1.2238
33	Cyclohexanone	C108941	C_6_H_10_O	98.1	893.3	387.113	1.1603
34	1-Hexanol	C111273	C_6_H_14_O	102.2	849.6	329.257	1.6519
35	(*Z*)-3-Hexen-1-ol	C928961	C_6_H_12_O	100.2	848.4	327.730	1.2479
36	(*E*)-2-Hexenal M	C6728263	C_6_H_10_O	98.1	842.2	320.354	1.1888
37	(*E*)-2-Hexenal D	C6728263	C_6_H_10_O	98.1	842.4	320.557	1.5448
38	2-Hexanone M	C591786	C_6_H_12_O	100.2	795.8	269.721	1.2009
39	2-Hexanone D	C591786	C_6_H_12_O	100.2	794.4	268.360	1.5006
40	Butyl formate	C592847	C_5_H_10_O_2_	102.1	734.8	212.077	1.2083
41	2-Acetylfuran	C1192627	C_6_H_6_O_2_	110.1	915.0	420.414	1.1273
42	Ethyl hexanoate	C123660	C_8_H_16_O_2_	144.2	1011.2	598.537	1.3476
43	2-Pentylfuran	C3777693	C_9_H_14_O	138.2	999.2	577.509	1.2509
44	Methyl 3-methylbutanoate	C556241	C_6_H_12_O_2_	116.2	773.4	247.497	1.1979
45	Butyrolactone D	C96480	C_4_H_6_O_2_	86.1	918.2	425.553	1.3043
46	Butyrolactone M	C96480	C_4_H_6_O_2_	86.1	920.3	428.935	1.0836
47	Propanal	C123386	C_3_H_6_O	58.1	542.3	109.322	1.1478

## Data Availability

The original contributions presented in the study are included in the article, further inquiries can be directed to the corresponding author.
